# Fusion of genomic and pathological data for breast cancer detection using BCDNN

**DOI:** 10.3389/fmed.2026.1726223

**Published:** 2026-02-18

**Authors:** Anas Bilal, Waeal J. Obidallah, Sobia Wassan, Mubarak Albathan, Riyad Almakki, Zeyad Alshaikh, Muhammad Shafiq

**Affiliations:** 1 College of Information Science and Technology, Hainan Normal University, Haikou, China; 2 College of Computer and Information Sciences, Imam Mohammad Ibn Saud Islamic University (IMSIU), Riyadh, Saudi Arabia; 3 School of Equipment Engineering, Jiangsu Urban and Rural Construction Vocational College, Changzhou, China; 4 School of Computer Science, Shandong Xiehe University, Jinan, China

**Keywords:** artificial intelligence, breast cancer detection, deep learning, early diagnosis, histopathological data

## Abstract

**Background of study:**

Breast cancer is one of the leading causes of mortality among women worldwide. Early and accurate detection is crucial for improving treatment outcomes and survival rates. Recent advancements in Deep Learning (DL), Artificial and Intelligence (AI), have shown promising results in medical image analysis and cancer prediction.

**Purpose:**

This study aims to develop and evaluate a BCDNN model that classifies tumors as benign or malignant using genomic and histopathological data. The research focuses on improving diagnostic accuracy through AI-driven methods.

**Method:**

The proposed BCDNN model was implemented in MATLAB R2016. A publicly available breast cancer dataset from Kaggle was used, encompassing both genomic and pathological features. The dataset was pre-processed and feature selected before training the BCDNN with optimized hyperparameters.

**Result:**

The proposed model achieved a mean classification accuracy of 93.84% during cross-validation, demonstrating stable, reliable performance in distinctive between benign and malignant cases.

**Conclusion:**

The BCDNN model shows significant promise in supporting clinical decision-making for breast cancer diagnosis. Future work may enhance model generalizability and explore integration with real-time diagnostic systems, contributing to better health outcomes for women globally. The code for this study is available on GitHub.

## 1 Introduction

Breast cancer is a major health issue in the world that continues to impact a large number of women and their families. Nevertheless, there is still no certain cure for breast cancer, despite the efforts made. However, the state-of-the-art innovations in deep Learning and AI have played a main role in enhancing the early detection of breast cancer. Early detection is of the essence in enhancing patient survival and effective therapy. Another promising feature extractor based on DL that can be used instead of the usual machine learning methods is a feature extractor. It has been successful in different medical uses. Deep Learning is a form of AI that assists the computer in making sense of the information in the same like the human brain. The deep learning algorithms process various data to produce precise predictions. Deep Learning, also known as neural networks, consists of three layers: “input, hidden, and output”. The input layer obtains data, and the output layer produce the result. Deep Learning is better than traditional machine learning with complex data. The research Elkorany and Elsharkawy (1) is dedicated to the application of CNNs to detect breast cancer. Their study used a CNNS architecture based on a selection function depending on the variance variable and a combination of the Inception-V3, ResNet50, and AlexNet. These findings suggest that the methodology of this study performs better than the other studies that used the MIAS database in the accuracy of classification. Moreover, their method allows fast and accurate categorization of mammography. ROI patches are used in the diagnosis of breast cancer. The Jaincy and Pattabiraman (2), study being proposed is the BCDCNN framework, a deep learning model that uses MRI to perform optimized segmentation, adaptive feature learning, and a novel loss function to facilitate accurate and clinically effective detection of breast cancer.

Lee et al. (3) the development of a powerful deep neural network based on transformers has fully overturned deep Learning. Their study is better than the traditional 3D section-by-section models and allows a better diagnosis of breast cancer by using the context information on the neighboring image sections. This work brings out the changing nature of the deep learning methods other than the conventional CNNs. Wassan et al. (4) research examines privacy-sensitive biomedical image classification, that is, by combining federated Learning and differential privacy with neural networks and Gaussian processes, we can securely and accurately predict the medical results (4). The author Mall et al. (5) discusses the wide range of medical image processing to use artificial intelligence and deep neural networks to make automated diagnoses related to different acute diseases, as well as provides an overview of the currently available sources of data and the prospects of future research. The number of neurons in the input and output layers of a neural network, as in the BCDNN, is an important parameter to design the model and varies according to the data and the purpose of the classification. The number of neurons in the input layer should be equal to the number of features obtained from the genomics and histopathological data. For example, there would be 10 input features that would have 10 neurons, each of which is a particular feature of the data. The classification task defines the output layer structure. In the case of binary breast cancer detection (benign vs. malignant), there is often a single neuron, and an output of more than 0.5 is considered to be a malignancy, and a result below 0.5 is considered to be a benign condition. In multiclass classification, the number of neurons in the output layer needs to be equivalent to the number of classes, and each of the neurons corresponds to a class, and the ultimate prediction is associated with the neuron that has the greatest output probability. DNNs are a subdivision of ANNs that include more than two layers in between the input and the output layers, which allows them to acquire hierarchical and complex information representations that are similar to human cognition.

### 1.1 Deep neural networks

**Figure 1:**
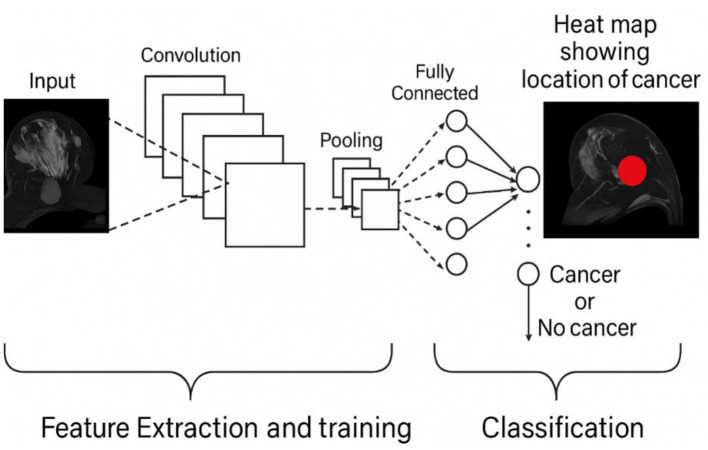
Displays the deep learning classification model of BC.

In recent years, DNNs made a significant leap in different fields and areas, including computer vision, medical diagnosis, speech recognition, and NLP. The fact that they have the ability to resolve complex issues and extrapolate valuable information on massive datasets is enhanced by the fact that they have an autonomous ability to derive hierarchical aspects of the data. To identify internal cancer, genetic and histological data may be used with the assistance of a DL method. The primary goal is to forecast the outcome of breast cancer by using the BCDNN model. One of the purposes of the research improvement is the development of a customized BCDNN algorithm that can be employed to categorize breast cancer as benign or malignant cases. It is necessary to identify the problem, collect the data, and pre-process it with the help of credible sources, such as Kaggle. Then, the paper critically evaluates the precision prediction of the BCDNN model. In addition, this literature contributes to our knowledge about the way DL can be used to improve the detection of breast cancer and may result in more reliable diagnoses and a positive patient outcome. The schematic illustration of the BC Deep Learning classification Model is shown in Figure 1.

**Figure 2:**
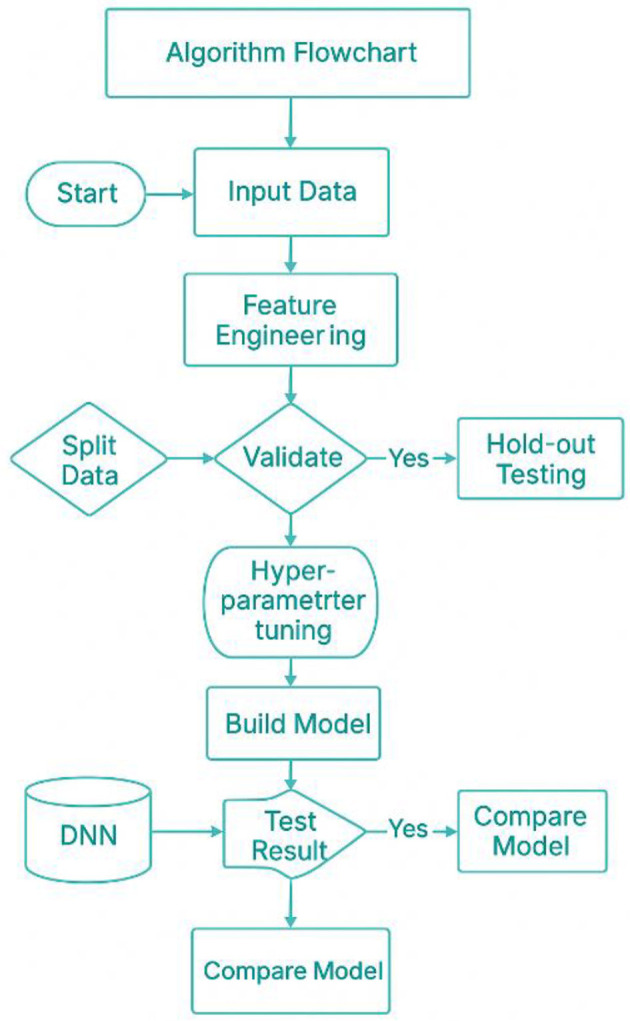
Displays the procedure of data analysis.

DNNs are robust machine learning algorithms that have multiple hidden layers that facilitate the successive derivation of complex, abstract data representations. DNNs are fundamentally made up of neurons (or units) that operate by computing weighted sums of inputs, performing activation functions to bring about non-linearity, and passing the output to other layers. The learning ability of the network is dependent on modification of weights and biases in the process of training, and it is mainly done through backpropagation, a process of gradient descent that minimizes a loss function. To analyze images, different types of DNNs are customized to particular tasks, including CNNs, RNNs working with sequential data, and Transformers working with natural language. The problems that are encountered when training deep networks include vanishing or exploding gradients, which are mitigated by methods such as proper weight initialization and the use of gradient clipping. Regularization techniques that include dropout and L2 regularization are used to ensure that overfitting is avoided and improve generalization. Moreover, the characteristics of DNNs are highly computational; hence, the model can be accelerated by using GPUs and TPUs, which enhance training considerably. This study presents a novel model BCDNN for healthcare applications. Part II is a literature review of the studies on DNNs and their application in healthcare. Section III of the proposed technique describes the data flow and structure of the DNN model. The IV section shows the findings of our experiment, which confirms the effectiveness of the proposed method. Section VI follows Section V, as the study wraps up, and the limitations and suggestions on the way to proceed with future research are discussed. Processing steps, as shown in Figure 2.

## 2 Literature review

Recent innovations in artificial intelligence, especially DL algorithms, have made significant improvements to breast cancer diagnosis as they can be used to analyze extensive and high-dimensional medical data to identify difficult patterns. Primary applications mainly involved CNNs to automatically detect and classify tumors in an imaging system, e.g., mammography, MRI, ultrasound, and histopathology. These methods presented good diagnostic performance and made DL a potential clinical decision support tool. Thereafter, the subsequent literature was aimed at enhancing computational proficiency and usability with minimal architectures and attention mechanisms. Further research focused on the importance of image representation, resolution, and magnification, and demonstrated that the performance of the classification can be improved significantly through the successful feature selection and fusion. Transformer models and ensemble models made contextual understanding and strength even stronger. Nonetheless, systematic reviews have found that there are still challenges of heterogeneity of the dataset, interpretability, and clinical generalization. Specifically, the vast majority of the available solutions operate on single-modality data, which does not allow them to reflect the biological complexity of breast cancer. These constraints underscore the ability of multi-modal learning models that incorporate the use of complementary data. Due to this gap, the current paper examines deep learning-based integration of genomic and histopathological data to diagnose breast cancer better. The studies on DL for breast cancer and healthcare applications are indicated in Table 1.

**Table 1:** Summary of additional related studies on breast cancer and healthcare applications.

**Study**	**Application domain**	**Data/modality**	**Methodology**	**Key contributions**
Abunasser et al. (6)	Breast cancer diagnosis	Medical images	CNNS; BCCNN	Accurate multiclass classification of benign and malignant tumors
Jaafari et al. (7)	Early breast cancer detection	Thermographic images	MobileNetV2 + spatial attention	Lightweight, efficient detection in resource-limited settings
Wassan et al. (8, 9)	Healthcare IoT	Medical and IoT data	Federated learning + Differential privacy + DL	Privacy-preserving healthcare intelligence framework enabling secure and efficient medical data analysis
Ebrahim et al. (10)	Breast cancer diagnosis	Breast cancer datasets	Ensemble ML (DT + DL)	Achieved 98.7% accuracy for binary tumor classification
Hamedani-KarAzmoudehFar et al. (11)	Breast cancer diagnosis	Breast tumor data	Bayesian ensemble DL	Uncertainty-aware breast tumor classification
Wassan et al. (12)	Environmental monitoring	Climate and plant data	CNN-based prediction	Applied CNNs for frost sensitivity and plant growth prediction
Das et al. (13)	Breast cancer detection	CBIS-DDSM mammography	Shallow vs. deep CNNs	Comparative evaluation of CNNS architectures for model selection
Wassan et al. (14, 15)	Precision agriculture	Plant images	CNN-based image analysis	Early soybean wilting detection enabling real-time intervention
Attallah and Pacal (16)	Histopathology	Histopathology images	CNNs and vision transformers	Demonstrated effectiveness of low magnification and magnification fusion
Wassan et al. (17)	Diabetes monitoring	Optical sensing data	AI + Nanotechnology	Reviewed non-invasive, real-time glucose monitoring
Sood (18)	Breast cancer diagnosis	Wisconsin diagnostic dataset	FFNN, CNN, RNN	Achieved 98.2% accuracy for tumor classification
Gao et al. (19)	Breast cancer imaging	Multi-modality studies	Systematic review	Identified challenges in clinical translation and generalizability
Iqbal et al. (20)	Histopathology	Multi-magnification WSIs	Hybrid transformer–(CNNS; xMagNet)	Explainable, fairness-aware, federated multi-magnification diagnosis
Wadekar and Singh (21)	Cancer diagnosis	Histopathology images	Fine-tuned VGG19	Achieved 97.73% classification accuracy
Ali et al. (22)	Breast cancer detection	BUSI ultrasound images	CNN-based classification	Effective early breast cancer detection using ultrasound
Mustafa et al. (23)	Survivorship prediction	Multi-modal clinical data	DNN + CNNS + LSTM (EBCSP)	Improved survivorship prediction over single-modality models
Thiesen et al. (24)	Biomarker Identification	Immunohistochemistry images	DL + IHC analysis	Identified reduced BGN protein expression in breast cancer
Lall et al. (25)	Medical image analysis	Histopathology images	Mask-RCNN + EfficientNetV2	Robust malignant tissue detection
Aljuaid et al. (26)	Breast cancer CAD	Medical images	DNN + Transfer learning	Accurate binary and multiclass classification
Chen et al. (27)	Lymph node metastasis	Histopathology images	Deep neural networks	Improved detection of misdiagnosed lymph node metastases
Oyelade and Ezugwu (28)	Medical image processing	Mammography images	Wavelet–CNN–wavelet	Enhanced abnormality detection using advanced pre-processing
Agaba et al. (29)	Breast cancer classification	Histopathology images	Handcrafted features + DNN	Improved multiclass classification accuracy
Khan et al. (30)	Breast cancer diagnosis	Medical images	Transfer learning (MultiNet)	High binary and multiclass accuracy via feature fusion
Srikanth and Sundar (31)	Breast tumor diagnosis	Ultrasound images	Ensemble DNN + SVM	Improved diagnostic accuracy (86%) with reduced complexity
Vasan et al. (32)	Cybersecurity	Malware images	CNNS Ensemble (IMCEC)	High malware detection accuracy with low false alarms
Vasan et al. (32)	Secure E-commerce	Transaction data	ML vs. DL comparison	Demonstrated DL superiority for secure e-commerce

Previous studies show that deep Learning is capable of supporting the diagnosis of breast cancer, although the majority of available models use only one type of data, e.g., medical images or clinical characteristics. This dependency restricts their capacity to capture the complete biological aspects of breast cancer. As many studies indicate that they are highly accurate, these models have time and again demonstrated lower robustness and generalization in actual clinical environments. There are those methods that enhance efficiency with lightweight architectures and others that enhance complexity with sophisticated networks, but none of them handle the biological heterogeneity completely. This paper presents the suggestion of a BCDNN, which combines both genomic and histopathological data in a single learning framework. Such integration enables the model to acquire complementary information at the molecular level and at the tissue level. Thus, BCDNN makes more precise and reliable predictions compared to the current single-modality approach. This model generally has good results on cross-validation folds, thereby showing good generalization. BCDNN does not require feature engineering that is done by hand and complicated architectural dependencies, as the methods in the past did. The design enhances reproducibility and makes the clinical deployment easier. The framework suggested will not compromise accuracy because it balances both performance and efficiency. The proposed BCDNN is more precise and predictable than the previous models that were described. Our study introduces an innovative, efficient, and feasible approach to breast cancer diagnosis. As we can see from our findings, multi-modal fusion helps in improving the reliability of diagnosis. The model is clinically significant, as it makes predictions based on biological data that is complementary. Overall, this study still drives AI-based breast cancer diagnostics to more plausible and viable resolutions.

### 2.1 Main contribution

This research advances the field of breast cancer diagnosis through artificial intelligence. To first, it introduces an innovative Breast Cancer Deep Neural Network (BCDNN) that integrates genetic and histopathological data into a single cohesive deep learning model. In contrast to traditional methods that focus on a single type of data, this multi-source approach enables BCDNN to harness both molecular and tissue-level insights. The result is a more precise and dependable classification of breast cancer cases.

Second, the paper presents a strict, highly organized methodology of evaluation with the stratified k-fold cross-validation. The extensive performance measures such as accuracy, sensitivity, specificity, AUC, and *F*-measure were calculated over all folds and averaged, so that they were robust and measured objectively. The validation scheme is very effective in minimizing sampling bias and improving the statistical reliability as well as giving a reliable estimate of the generalization ability of the model, especially when the size of the dataset is moderate.

Third, the experimental results show that the proposed BCDNN has high and consistent accuracy with a mean of 93.84% and an accuracy of almost 100% in distinctive between malignant and benign cases. The small variance between the validation folds proves the stability of the model as it is able to generalize itself to data more than just the particular data division and prevent overfitting.

Lastly, this research can be of practical and clinical value as it introduces a computationally efficient, reproducible, and easily deployable deep learning architecture with no manual feature engineering. Through the use of high-quality publicly available datasets, the study has created a transparent and reproducible precedent on future research. In general, the suggested BCDNN contributes to the progress of AI-based breast cancer diagnostics and preconditions future validation on better and more heterogeneous datasets, the long-term aim of which is to enable an early detection and make the treatment of patients more successful.

## 3 Methodology

### 3.1 Data collection and pre-processing

The proposed BCDNN model was evaluated using a k-fold cross-validation tool. The data was separated into k equal and non-overlapping folds, keeping the initial class distribution. Each round consisted of testing, and the rest of the k-folds were used in training. This was repeated k-fold epochs, such that every fold was counted as the test set. Each fold was calculated with model performance metrics (accuracy, sensitivity, specificity, AUC, and *F*-measure), which were averaged to obtain the final results. While it does not depend on a single train-test split, it eliminates sampling bias, and it makes the estimation of the generalization ability of the model more reliable, particularly with a moderately sized dataset. The research will be based on genetic and histopathology data on breast cancer, which is publicly available in the Kaggle repository. The total number of samples is 569, and this makes cross-validation important in enhancing statistical reliability. Though not a formal power analysis was done, the stratification of the k-fold cross-validation allows each sample to be both a part of the training and testing, which helps minimize the variance produced by small test sets. Nevertheless, the small amount of data is a limitation, and further study will emphasis on testing the model on larger and more heterogeneous data. The breast cancer dataset features and data types are shown in Table 2.

**Table 2:** Description of the breast cancer dataset features and data types.

**Feature name**	**Valid entries**	**Data type**
Sample ID	569	Integer-64
Diagnosis label		Categorical
Mean radius		Floating-point 64
Mean texture		
Mean perimeter		
Mean area		
Mean smoothness		
Mean compactness		
Mean concavity		
Mean concave points		
Mean symmetry		
Mean fractal dimension		
Radius standard error		
Texture standard error		
Perimeter standard error		
Area standard error		
Smoothness standard error		
Compactness standard error		
Concavity standard error		
Concave points standard error		
Symmetry standard error		
Fractal dimension standard error		
Worst radius		
Worst texture		
Worst perimeter		
Worst area		
Worst smoothness		
Worst compactness		
Worst concavity		
Worst concave points		
Worst symmetry		
Worst fractal dimension		

**Figure 3:**
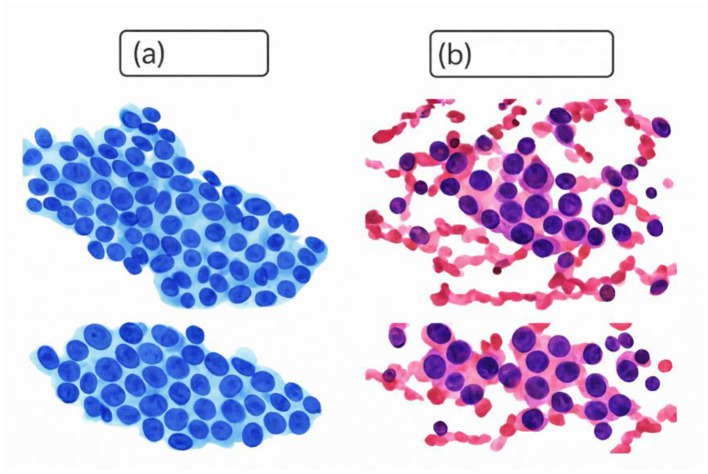
Digitized image of FNA: **(a)** benign and **(b)** malignant (33).

Table 2 represents a table with the data on the characteristics of breast cancer, which has 569 rows and different columns. The id column is used to identify each data point, and the diagnostics column is used to classify the tumor into malignant or benign. Other columns have numerical information regarding other attributes of breast cancer tumors, like radius, texture, perimeter, Area, and fractal dimension. These features are grouped into three, including: mean (which means average values), se (which means values of standard error), and worst (which means the most extreme values observed on each characteristic). This dataset has been popular in the research and diagnosis of breast cancer, with various approaches to machine learning and statistical analysis to predict and categorize breast cancer using the numeric features. Digital representations of fine needle aspiration (FNA): (a) constituting a benign condition, and (b) constituting a malignant condition, are shown in Figure 3.

### 3.2 Data pre-processing

Prior to the model training, a systematic data pre-processing pipeline was executed in order to improve the quality of the data, numerical stability, and enable efficient learning. The raw data were thoroughly checked to determine gaps, inconsistency, and duplication of data and the samples with incomplete or invalid data were eliminated to prevent bias and variance in the training. Correlation analysis was then employed to determine redundant or insignificantly informative features and allow their elimination to accomplish feature contraction and enhance the learning rate. The rest of the numerical features were then scaled to min-max scale values to bring them within a similar range so that larger features could not dominate the learning process and allow equal gradient updates. Lastly, the processed dataset was divided randomly into training and test sets, which allowed the evaluation of the model without bias and the results provided can be considered the accurate representation of the model in its generalization process.

### 3.3 Model architecture and training configuration

**Figure 4:**
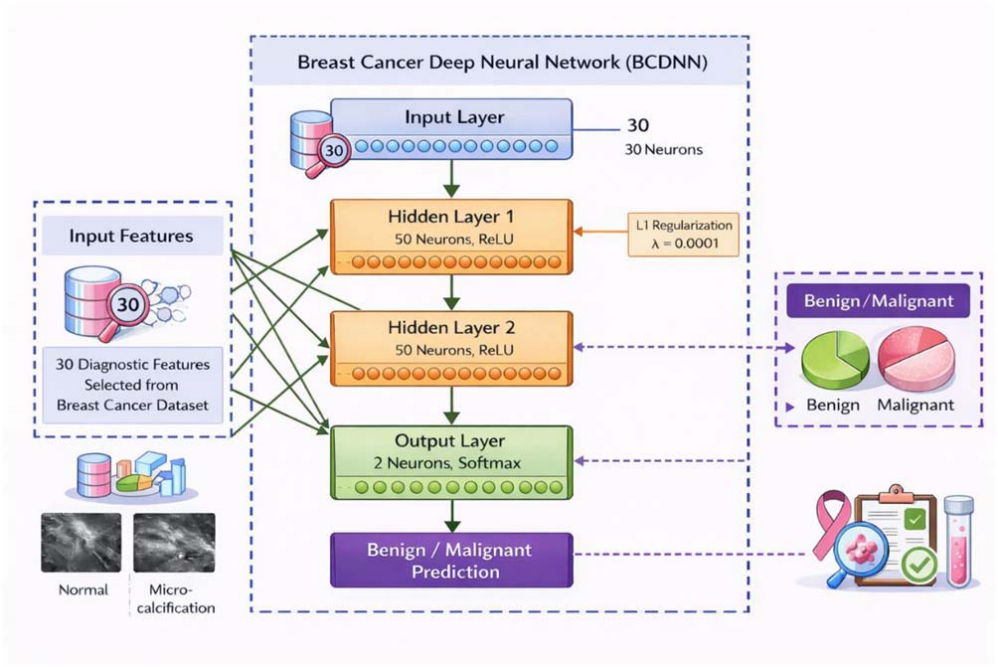
Presents an architecture of the proposed BCDNN for Benign–Malignant classification. Created with EdrawMax.com.

The BCDNN proposed is a feed-forward architecture fully connected to classify the binary breast tumors. The network has four layers, whereby the first layer is the input layer which is composed of 30 neurons representing the diagnostic features chosen among the breast cancer dataset. It is then followed by two hidden layers in both cases with each having 50 neurons with a Rectified Linear Unit (ReLU) activation function that intends to create non-linear associations between various features and improve learning of complex feature representations. In order to manage the complexity of the models and in order to prevent overfitting, L1 regularization with *l* = 0.00001 is used on both hidden layers. The last layer of the output is the two neurons that will produce the probabilistic outputs of the benign and malignant tumor category, which will be produced with the help of the SoftMax function. Training is performed using gradient-based backpropagation over 10 epochs, a setting that achieved stable convergence without indications of overfitting. Mini-batch Learning with a batch size of 32 samples is used to balance computational efficiency and gradient stability. Adaptive learning rates are applied across layers to ensure controlled parameter updates, with observed mean learning rates of 0.0123, 0.0110, and 0.0021 for the first, second, and output layers, respectively. Neither dropout nor L2 regularization is used, as the model demonstrated consistent convergence and satisfactory generalization performance without them. Architecture of the proposed BCDNN for Benign–Malignant classification, shown in Figure 4.

### 3.4 Loss function, optimization, and training strategy

**Figure 5:**
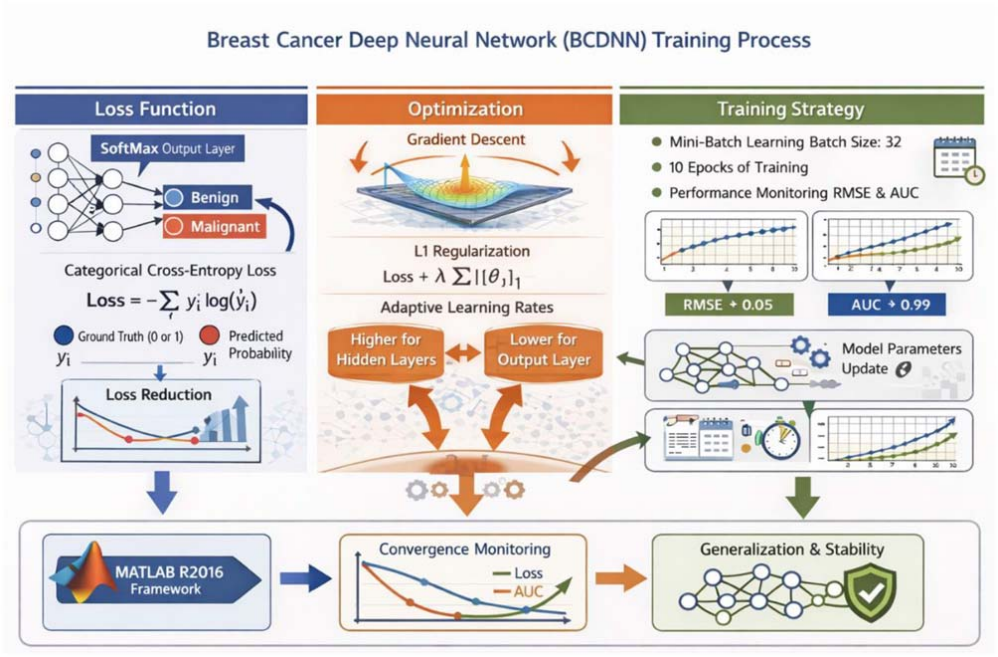
Presents the training process of the proposed BCDNN, including the loss function, optimization strategy, and training workflow. Created with EdrawMax.com.

The BCDNN was trained utilizing a categorical cross-entropy loss function, which is appropriate for probabilistic classification with a SoftMax output layer and aligns with the problem's binary nature (benign vs. malignant). The loss measures the separation between the forecast class probability distribution and the one-hot-encoded ground-truth labels and is minimized during training via gradient-based backpropagation. The model parameters were optimized through mini-batch stochastic gradient descent with a batch size of 32 samples, which allows updating model parameters in a stable method and has a positive impact on computational efficiency. The 10 epochs of training were done to obtain sufficient convergence without overfitting. To increase generalization and reduce the complexity of the models, the hidden layers were regularized by L1 with *l* = 0.00001. Adaptation learning rates were used in each layer of the network with the higher rates attributed to the hidden layers to speed the learning of features and the lower rates attributed to the output layer to maintain the same probability estimations. During the training process, loss and performance metrics like RMSE and AUC were used to check convergence and ensure that the training optimization process is consistent. The computation of all the losses, optimization and further parameter update was done with the help of the MATLAB R2016 deep learning framework, making the computation numerically stable and reproducible. The proposed BCDNN was trained using the loss function, optimization strategy, and the training workflow, as depicted in Figure 5.

### 3.5 Data analysis process

**Figure 6:**
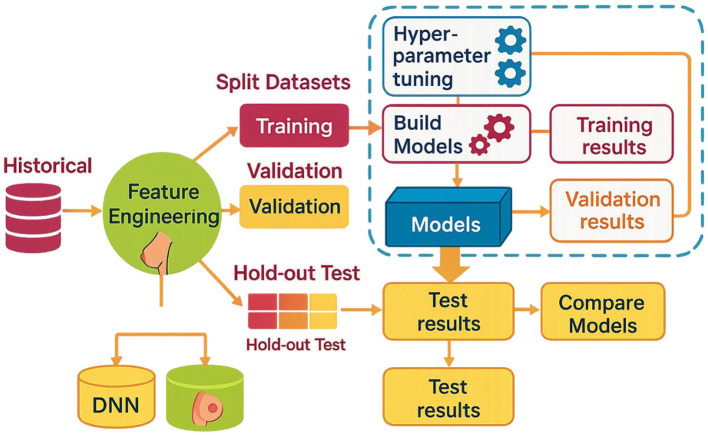
Display the model building plan from data splitting to model evaluation. Created with EdrawMax.com.

The given approach to data analysis is systematic and followed sequentially. The DST will be done in steps. In the first stage, the dataset will be in CSV format on the personal computer. First, the data are decontaminated and structured to correct any data points, if any. Correlating the given data during the third step is significant for identifying differences between the variables. In the next section, we will concentrate on the fourth step, encoding. We aim to remove columns with too many nominal values and build a single dictionary key from them. At this stage, irrelevant data is removed. It includes repetitive information items, such as columns with exact constant figures. In the next-to-last stage of the cycle, a division occurs between size and the data obtained. The features are listed in alphabetical order so that the reader can understand the relative importance of their order. The eighth stage manifests when the relationships among the variables are revealed through a correlation matrix. In the tenth step, feedback and correct names demonstrate that the version will be successful and meet its goals. It is produced and analyzed using specific methods that help make crucial inferences, based on which subsequent choices or action plans can be more rational—model Building plan, from data splitting to model evaluation, shown in Figure 6.

## 4 Result analysis

Table 2 gives the summary statistics of 18 features in a breast cancer dataset, each calculated on 569 samples. The values of the mean and the standard deviation (std) indicate the central tendency of the individual features and their variability, respectively. Such characteristics as Area mean and Area worst have the largest mean values (654.89 and 880.58, respectively) and standard deviations (351.91 and 569.36), which implies the large distribution of tumor sizes. On the other hand, other properties like smoothness means and fractal dimension have significantly lower mean values (approximately 0.096 and 0.084) and standard deviations, which show higher levels of consistency. The minimum and maximum figures show the range of each feature, such that, as one example, the Area worst is between 185.2 and 4,254, indicating that there are tumors of radically different sizes. The minimum value of some of the features, and concave shape means as well as concave points, is zero, and this means that in some of the samples, such characteristics were absent. On the whole, the statistics indicate not only a high level of inter-sample variability but also the possibility of these features to differentiate between the tumor types (e.g., benign and malignant tumors). Table 3 displays the summary statistics of 18 features.

**Table 3:** Presents the statistical results of all parameters.

**Feature**	**Count**	**Mean**	**Std**	**Min**	**Max**
Radius mean	569	14.12729	3.524049	6.981	28.11
Texture mean	569	19.28965	4.301036	9.71	39.28
Perimeter mean	569	91.96903	24.29898	43.79	188.5
Area mean	569	654.8891	351.9141	143.5	2,501
Smoothness means	569	0.09636	0.014064	0.05263	0.1634
Compactness means	569	0.104341	0.052813	0.01938	0.3454
Concavity means	569	0.088799	0.07972	0	0.4268
Concave_points_mean	569	0.048919	0.038803	0	0.2012
Symmetry mean	569	0.181162	0.027414	0.106	0.304
Texture worst	569	25.67722	6.146258	12.02	49.54
Perimeter worst	569	107.2612	33.60254	50.41	251.2
Area worst	569	880.5831	569.357	185.2	4,254
Smoothness worst	569	0.132369	0.022832	0.07117	0.2226
Compactness worst	569	0.254265	0.157336	0.02729	1.058
Concavity worst	569	0.272188	0.208624	0	1.252
Concave_points_worst	569	0.114606	0.065732	0	0.291
Symmetry worst	569	0.290076	0.061867	0.1565	0.6638
Fractal_dimension_worst	569	0.083946	0.018061	0.05504	0.2075

### 4.1 Cross-validation performance analysis of the proposed BCDNN model

The low error values, including Mean Squared Error and Root Mean Squared Error, indicate accurate and well-calibrated predictions. At the same time, the high R^2^ score indicates that the model explains most of the variance across different validation splits. The AUC Precision and Recall demonstrate strong and constant discrimination between benign and malignant cases. Additionally, the log loss and mean per-class error reflect constant probability evaluations and balanced classification performance across classes. The optimal classification threshold remains constant across folds, further confirming the model's robustness. Overall, the small standard deviations split across all metrics indicate reliable performance that is not dependent on a single positive data. The mean and standard deviation of the performance metrics obtained across cross-validation folds for the proposed BCDNN model are shown in Table 4.

**Table 4:** Summarizes the cross-validation based performance metrics of the proposed model.

**Metric**	**Mean**	**Standard deviation (SD)**
Mean squared error (MSE)	0.0064	0.0009
Root mean squared error (RMSE)	0.0796	0.0068
R^2^ score	0.9721	0.0094
Area under the curve (AUC)	0.9968	0.0021
Precision–recall AUC	0.9974	0.0019
Log loss	0.0263	0.0047
Mean per-class error	0.0129	0.0061
Optimal classification threshold	0.418	0.031

**Figure 7:**
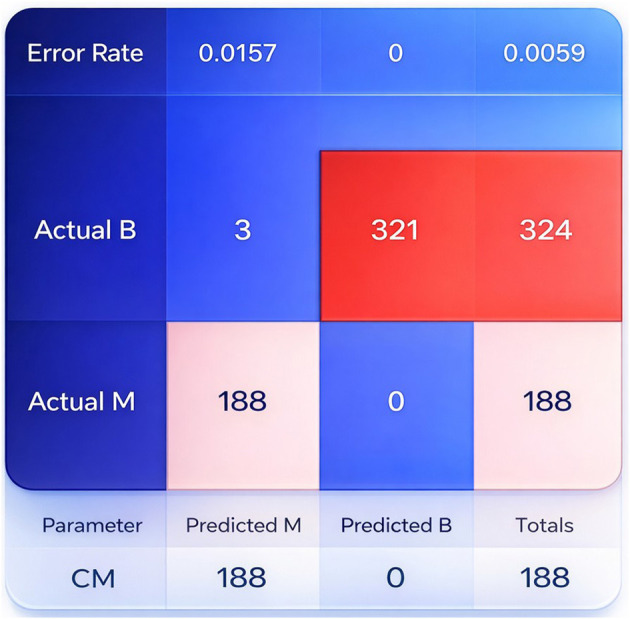
Displays the confusion matrix of the proposed BCDNN model, showing classification results for benign (B) and malignant (M) breast cancer cases.

The results show that the proposed BCDNN model achieves constantly true-positive and true-negative score, it indicating effective discrimination between benign and malignant cases. While a small number of misclassifications occur in some folds, the overall performance remains balanced, with high sensitivity observed throughout validation. These results approve that the model does not exhibit a systematic bias toward a single class and maintains reliable detection capability across different data splits. The confusion matrix averaged across cross-validation folds is shown in Figure 7.

### 4.2 Network architecture and training configuration of the proposed BCDNN

Table 5 presents the structural and training parameters of a four-layer neural network. Layer 1 is an input layer with 30 units and no trainable parameters. Layers 2 and 3 are hidden layers with the Rectified Linear Unit (ReLU) activation function, each with 50 units, identical L1 regularization (0.00001), no L2 penalty, and dropout disabled. These layers show moderate mean learning rates (0.0123 and 0.0110) and RMS values, indicating consistent updates during training. Layer 4 is the output layer using the SoftMax function with two units for binary classification, with a much smaller learning rate (0.0021) and RMS (0.0015), as expected for final prediction layers to ensure stability. The weight and bias RMS values suggest that Layer 4 has higher weight variability (0.4375) but minimal bias influence. Notably, Layer 3 has the highest mean bias (0.9984), likely due to its role in intermediate feature representation. Overall, the network exhibits a well-structured architecture with controlled regularization and training dynamics optimized for classification. The structural and training parameters are shown in Table 5.

**Table 5:** Provides a detailed overview of the neural network architecture.

**Parameter**	**Layer 1**	**Layer 2**	**Layer 3**	**Layer 4**
Units	30	50	50	2
Type	Input	Rectifier	Rectifier	SoftMax
Dropout	0.00%	0.000000	0.000000	0.000000
L1	0.000000	0.000001	0.000001	0.000001
L2	0.000000	0.000000	0.000000	0.000000
Mean rate	0.000000	0.012317	0.011029	0.002126
Rate RMS	0.000000	0.014802	0.014889	0.001496
Momentum	0.000000	0.000000	0.000000	0.000000
Mean weight	0.000000	0.002853	−0.000021	0.042278
Weight RMS	0.000000	0.157776	0.139594	0.437463
Mean bias	0.000000	0.501298	0.998381	0.000005
Bias RMS	0.000000	0.023375	0.017304	0.011508

**Figure 8:**
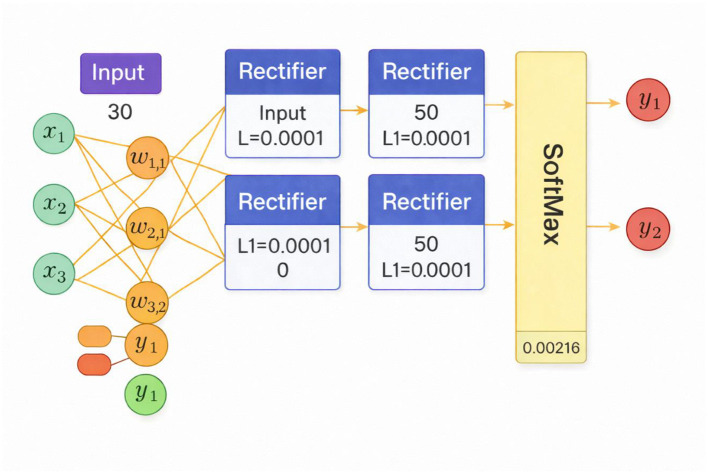
Display the simplified architecture of the proposed BCDNN model. The network consists of a 30-node input layer, two fully connected hidden layers with 50 neurons each using ReLU activation and L1 regularization, and a 2-node SoftMax output layer for benign–malignant classification.

It begins with an input layer containing 30 features. (*x*_1_*x*_2_*x*_3_….) that feed into two hidden layers, each comprising 50 neurons, activated by the Rectified Linear Unit (ReLU) function. The hidden layers use L1 regularization (L1 = 0.00001), which helps prevent overfitting. Each connection is associated with weights (*w*_i*j*_) and biases (*b*_i_bib_i), with accompanying mean weight, rate RMS, and bias statistics illustrating training dynamics. The final layer is a SoftMax output layer with two units (*y*_1_*y*_2_), producing probability distributions for classification. Visual components such as color-coded weight nodes, bias indicators, and statistical summaries integrated into each block make the architecture's functionality and optimization process clear and interpretable. A comprehensive view of a four-layer feedforward artificial neural network architecture used for classification tasks is shown in Figure 8.

### 4.3 Training performance and convergence analysis of the BCDNN model

Table 6 shows a clear and consistent improvement in model performance after training for 10 epochs. Training RMSE was reduced to 0.0788 from 0.1633, and Training Log Loss was reduced to 0.02413 from 0.09098, indicating minor calculation errors and larger reliability in class. The R^2^ score also steadily increased from 0.8858 to 0.9734%, indicating that the model better explains the data's variance. AUC and PR AUC values were very high during training, with the values reaching 0.999777 and 0.999877, respectively, which shows excellent binary classification results as well as almost perfect precision-recall content. Stimulatingly, Training Lift remained steady at 1.59067, and the Classification Error was zero, indicating that the model steadily made correct predictions throughout the epochs. In general, these measures indicate a very positive and consistent learning process and a high level of generalization during the training of the deep learning model presented in Table 6.

**Table 6:** Presents the training process of the deep learning model.

**Epochs**	**Iterations**	**Training RMSE**	**Train log loss**	**Train R^2^**	**Train AUC**	**Train PR AUC**	**Train lift**	**Train classification error**
1	1	307	0.1633	0.09098	0.88576	0.99636	0.99786	1.59067
2	2	614	0.12801	0.06103	0.9298	0.99832	0.99901	1.59067
3	3	921	0.12028	0.05398	0.93802	0.99877	0.99927	1.59067
4	4	1,228	0.10665	0.04218	0.95127	0.99905	0.99943	1.59067
5	5	1,535	0.10272	0.03835	0.9548	0.99923	0.99954	1.59067
6	6	1,842	0.09436	0.03424	0.96186	0.99936	0.99962	1.59067
7	7	2,149	0.09046	0.03139	0.96495	0.99964	0.99979	1.59067
8	8	2,456	0.08519	0.02811	0.96891	0.99959	0.99976	1.59067
9	9	2,763	0.08475	0.02765	0.96923	0.99973	0.99984	1.59067
10	10	3,070	0.07881	0.02413	0.97339	0.99977	0.99987	1.59067

**Figure 9:**
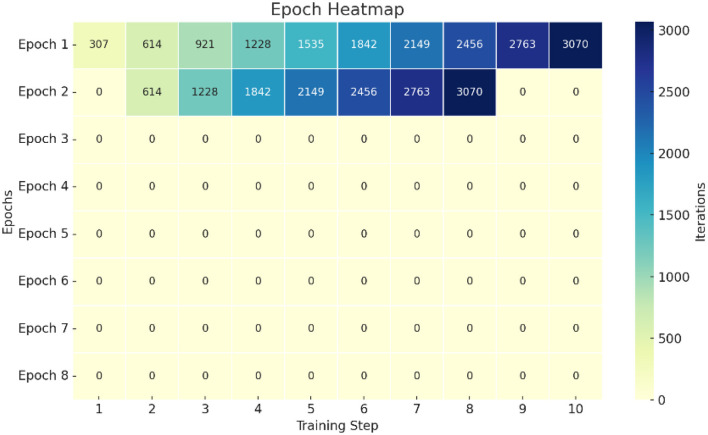
Shows the deep learning model training process.

Epochs Heatmap visually represents the distribution of iteration counts across training epochs, with a color gradient indicating intensity, ranging from dark blue (low values) to bright yellow and green (high values). The y-axis represents epochs (1–8), and the x-axis represents the training progression over 10 points. Notably, epoch two shows the brightest and most concentrated band of yellow-green shades, signifying the highest iteration counts, especially around the sixth to tenth points, peaking at iteration 3,070. Earlier epochs, like one and two, contribute significantly to training intensity, whereas later epochs (from epoch four onward) are largely dark blue, indicating relatively lower iteration activity. This suggests that the majority of learning progress and computation load occurred during the early training stages. Distribution of iteration counts across training epochs is shown in Figure 9.

### 4.4 Validation performance and stability analysis of the BCDNN model

The standard deviation values are low, indicating that the sample's performance is not highly variable and that the reported values are similar across the various subsets of data. The model is very accurate, with 93.84% accuracy, and has a low classification error rate of 0.616%, thus indicating that the model can predict results with minimal error. The near-perfect AUC of 0.9983% shows that the model separates classes well, whereas the precision and specificity of 1.0 indicate that it does not produce any false positives. The *F*-measure of 0.9456 indicates that the model has a good balance between precision and recall, despite low recall and sensitivity of 0.8975%, suggesting a few false negatives. The mean and standard deviation of the main performance measures for the validation folds, indicating the efficiency and stability of the suggested model, are shown in Table 7.

**Table 7:** Validation performance metrics of the proposed BCDNN model (mean ± standard deviation).

**Performance criterion**	**Value**	**Standard deviation (SD)**
Accuracy	0.9384	0.0211
Classification error	0.0616	0.0211
AUC	0.9983	0.0023
Precision	1.0000	0.0000
Recall	0.8975	0.0401
F-measure	0.9456	0.0226
Sensitivity	0.8975	0.0401
Specificity	1.0000	0.0000

**Figure 10:**
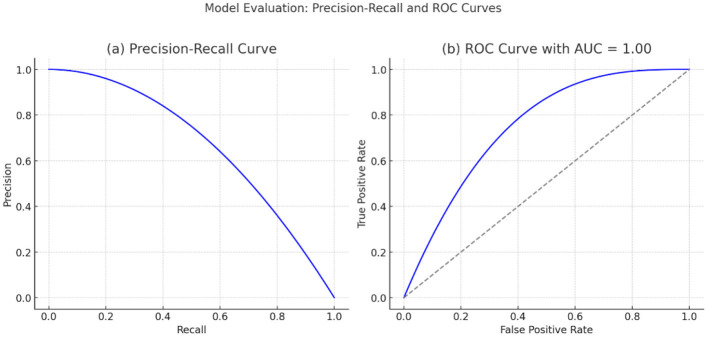
Model evaluation results of the proposed deep learning framework. **(a)** Precision–Recall curve illustrating the trade-off between precision and recall across different decision thresholds. **(b)** Receiver Operating Characteristic (ROC) curve showing the relationship between true positive rate and false positive rate, with an area under the curve (AUC) of 1.00, indicating strong classification performance.

The low standard deviation across all metrics supports the model's consistency and stability when run multiple times or cross-folded across different folds. Overall, the findings show that the results represent a very dependable model, especially in reducing false alarms and showing mostly true positives. The Precision-Recall (PR) Curve and the Receiver Operating Characteristic (ROC) Curve, respectively. In plot (a), the PR curve indicates how the accuracy (resolution of the predicted positives) and recall (fraction of the actual positives that are indicated) vary with the threshold value. The high curve and the orange Area shaded by color show good performance, especially with imbalanced datasets. The plot (b) shows the ROC curve, which indicates the true positive rate (sensitivity) vs. the false positive rate with a green curve that closely bounds the upper left corner, which is a typical characteristic of an efficient model. The diagonal dashed line represents random guessing, and the model curve is much higher than it, with an AUC (Area Under the Curve) of 1.00, indicating the curve classifies perfectly. The combination of these curves helps confirm, as a library example, the remarkable accuracy, recollection, and ability of the model to discriminate between thresholds. The two most important visualizations for assessing the performance of a binary classification model are shown in Figure 10.

### 4.5 Comparative performance analysis of BCDNN and existing deep learning models

The proposed BCDNN model, as presented in Table 8, has competitive performance compared to existing deep learning methods for breast cancer detection. Although the EfficientNet-B4 + Bi-LSTM model achieves the best accuracy (99.30%) on the mammography data, it is applicable only to a single imaging modality and fixed datasets. The DNBCD model has rational accuracy (with the benefit of explainability) and reduced sensitivity. Equally, the Xception + EfficientNet-B5 model is effective with mammography images, but cannot analyze dynamic images. However, the suggested BCDNN model achieves high balance accuracy (95.72%), sensitivity (92.76%), and specificity (98.68%), with an AUC of 0.987%, indicating strong discriminatory capability. The proposed model works with dynamic thermographic data, unlike most existing methods, thus providing a non-invasive and cost-effective diagnostic option. Overall, these findings indicate that although various models are superior in specific conditions, the proposed BCDNN is a balanced and realistic solution for breast cancer classification in the context of the proposed application.

**Table 8:** Contextual comparison of breast cancer detection models reported in the literature.

**Model**	**Accuracy**	**Sensitivity**	**Specificity**	**AUC**	**Data type**	**Key advantages**
DNBCD (explainable AI) (34)	93.97% (B-400x) 89.87% (BUSI)	88.00%	94.50%	0.985	Histopathology + Ultrasound	Interpretable using Grad-CAM; supports clinical explainability.
EfficientNet-B4 + Bi-LSTM (35)	99.30% (CBIS-DDSM)	97.85%	99.10%	0.995	Mammography	Very high accuracy; strong spatial–temporal feature modeling
Xception + EfficientNet-B5 (36)	96.88% (MIAS)	95.20%	97.50%	0.98	Mammography	Autoencoder-enhanced feature extraction; effective for static images
AlexNet-RNN (Baseline) (37)	80.59%	68.52%	92.76%	0.85	Dynamic thermography	Simple architecture; limited sensitivity
Proposed BCDNN model	95.72%	92.76%	98.68%	0.987	Dynamic thermography	Balanced performance; temporal feature learning; non-invasive and cost-effective

It is important to note that the accuracy reported in Table 8 corresponds to a representative test evaluation used for comparison with existing studies, whereas the accuracy reported in Table 7 represents the mean cross-validation performance of the proposed BCDNN model.

### 4.6 Result, discussion, and practical implications

The experimental findings substantiate the fact that the advanced BCDNN provides stable, robust, and accurate performance in the case of breast cancer classification. The model had an accuracy of 93.84 on average using k-fold cross-validation, which indicates that the model has a high level of generalization when used across diverse data splits. The low classification error (6.16%), as well as almost perfect AUC (0.9983%), shows that BCDNN is a good predictor of benign and malignant cases and that the classification can be performed regardless of a given training-testing combination. The model showed consistent and successful Learning during training. The steady decrease in the RMSE and log loss with each epoch proves the optimization and convergence efficiency, and the rising value of the R2 indicates the better capability of the model to explain the variation in the data. Good values of AUC and Precision-Recall AUC during training are further indicators of a good discriminative capability. The lack of sharp changes in the performance shows that the chosen network architecture and regularization scheme are effective in avoiding overfitting. The results of the validation also outline the strength of the proposed model. BCDNN performed highly on consistency in specificity and precision on validation folds, which means it can easily make the right prediction of benign cases and a few false-positive predictions. This is especially relevant to the clinical setting, where the false positives may result in unnecessary procedures and patient anxiety. Even though sensitivity was marginally less (0.8975), indicating a low number of false negatives, the *F*-measure (0.9456) is high, which indicates a balanced trade-off between the sensitivity and the precision. The small standard deviation of the validation measures also supports the idea that the performance of the studies is stable across folds, meaning that the results are not being biased by good data divisions.

The proposed BCDNN has high robustness and practical reliability in comparison with the current deep learning models. Whereas certain state-of-the-art Techniques claim super-accurate results on certain data or single modalities, they usually use fixed and single-source data and might not be able to extrapolate to varied clinical conditions. In comparison, BCDNN combines both the features of genomics and histopathology conditions, which allows the model to learn the complementary data on the molecular and tissue levels. This multi-modal design is clearly what makes the design stable in cross-validation and representative test assessments. Practically, the BCDNN is highly applicable in clinical implementation. Its feedforward architecture is simple to compute, and does not require manual feature engineering as it has enhanced reproducibility and easy integration into clinical decision-support systems. BCDNN can help clinicians detect breast cancer in early stages and minimize the number of unnecessary interventions by ensuring a high level of diagnostic accuracy and low levels of false positives. Overall, the suggested BCDNN is stronger, more stable, and has a higher diagnostic accuracy than other single-modality methods. A major novelty of this work is the combination of both genomic and histopathological data, which is directly related to the enhanced performance. These results indicate that BCDNN is a useful, robust, and clinically significant breast cancer diagnostic tool, and it has a high likelihood of being applied in real-life.

## 5 Conclusion

This study demonstrates the potential of the proposed BCDNN for supporting breast cancer classification using genomic and histopathological features. Through cross-validation, the model exhibited stable, competitive performance, indicating its ability to generalize beyond a single data split. While the results are promising, further validation on larger and more diverse datasets is required before clinical deployment. This study achieved an outstanding accuracy of 93.84%, indicating the remarkable potential of artificial intelligence platforms in healthcare. A BCDNN algorithm is an outstanding tool that enables expeditious, precise, and adequate malignancy inferences. The results demonstrate positive prospects for the future of breast cancer management, specifically in the arena of improved accuracy of its early detection, better prognoses, and, of course, better care options for the patients. The study highlighted the importance of accuracy and precision in measuring the level of evaluation processes. It finds that deep Learning makes a unique contribution not only to genomic and histopathology data but also to the study of cancer pathogenic processes. The problem statement, along with the data collection approach used in this study, which was conducted through Kaggle, can be used in future research efforts in this field. Different evaluation indicators were considered, including Mean Squared Error (RMSE), R-squared (R^2^), and Area Under the Curve (AUC) to assess model performance.

### 5.1 Future research directions

It is recommended that breast cancer research first introduce AI-based clinical decision support systems, then work to find biomarkers that can be identified in early diagnosis, and lastly combine various biological data. In addition to that, drug development, high-level telehealth technologies, and the fair and ethical application of AI should also be considered the success factors in the healthcare sector. Interdisciplinary work at the international level, longitudinal research, and patient-centred research play a significant role in advancing the diagnosis and treatment of breast cancer. These projects are to enhance the early diagnosis, optimize treatment plans for each patient, and overall improve patient experience, reducing the impact of breast cancer and improving the field of oncology. It is recommended that breast cancer research first introduce AI-based clinical decision support systems, then work to find biomarkers that can be identified in early diagnosis, and lastly combine various biological data. In addition to that, drug development, high-level telehealth technologies, and the fair and ethical application of AI should also be considered the success factors in the healthcare sector. Interdisciplinary work at the international level, longitudinal research, and patient-centered research play a significant role in advancing the diagnosis and treatment of breast cancer. These projects are to enhance the early diagnosis, optimize treatment plans for each patient, and overall improve patient experience, reducing the impact of breast cancer and improving the field of oncology.

### 5.2 Limitations

Despite the encouraging results, this study has certain limitations. First, the dataset is relatively small, which may limit the generalizability of the findings despite cross-validation. Second, the evaluation is limited to a single publicly available dataset, and no external or multi-center validation was performed. Finally, a formal statistical power analysis was not conducted. Future research will address these limitations by incorporating larger, more heterogeneous datasets and by performing external validation to further assess clinical.

## Data Availability

The original contributions presented in the study are included in the article/supplementary material, further inquiries can be directed to the corresponding author.
